# An immune-related lncRNA signature predicts prognosis and adjuvant chemotherapeutic response in patients with small-cell lung cancer

**DOI:** 10.1186/s12935-021-02357-1

**Published:** 2021-12-20

**Authors:** Zhihui Zhang, Yuejun Luo, Chaoqi Zhang, Peng Wu, Guochao Zhang, Qingpeg Zeng, Lide Wang, Liyan Xue, Zhaoyang Yang, Hua Zeng, Bo Zheng, Fengwei Tan, Qi Xue, Shugeng Gao, Nan Sun, Jie He

**Affiliations:** 1 Department of Thoracic Surgery, National Cancer Center/National Clinical Research Center for Cancer/Cancer Hospital, Chinese Academy of Medical Sciences and Peking Union Medical College, Beijing, 100021 China; 2 State Key Laboratory of Molecular Oncology, National Cancer Center/National Clinical Research Center for Cancer/Cancer Hospital, Chinese Academy of Medical Sciences and Peking Union Medical College, Beijing, 100021 China; 3 Department of Pathology, National Cancer Center/National Clinical Research Center for Cancer/Cancer Hospital, Chinese Academy of Medical Sciences and Peking Union Medical College, Beijing, 100021 China

**Keywords:** Small cell lung cancer, Immune response, lncRNAs, Chemotherapy, Individualized medicine

## Abstract

**Background:**

Patients with small-cell lung cancer (SCLC) are burdened by limited treatment options and the disease’s dismal prognosis. Long non-coding RNAs (lncRNAs) are essential regulators of genetic alteration and are actively involved in tumor immunity. However, few studies have examined interactions between immune genes and lncRNAs in SCLC.

**Methods:**

Immune-related lncRNA (irlncRNA) expression profiles and their clinical significance were explored. We enrolled 227 patients with SCLC, including 79 cases from GSE65002 and 148 cases from a validation cohort with corresponding qPCR data. The least absolute shrinkage and selection operator (LASSO) model was applied to identify prognostic irlncRNAs for an irlncRNA-based SCLC signature. We additionally investigated the potential mechanisms and immune landscape of the signature using bioinformatics methods.

**Results:**

An irlncRNA signature including 8 irlncRNAs (ENOX1-AS1, AC005162, LINC00092, RPL34-AS1, AC104135, AC015971, AC126544, AP001189) was established for patients with SCLC in the training cohort. Low-risk patients were more likely to benefit from chemotherapy and achieve a favorable prognosis. The signature was also well-validated in the validation cohort and various clinical subgroups. Compared to other clinical parameters, the irlncRNA signature exhibited superior predictive performance for chemotherapy response and prognosis. The signature was as an independent prognostic factor in the training and validation cohorts. Interestingly, low-risk patients showed an activated immune phenotype.

**Conclusion:**

We constructed the first irlncRNA-based signature for chemotherapy efficacy and outcome prediction. The irlncRNA signature is a reliable and robust prognostic classifier that could be useful for clinical management and determination of potential chemotherapy benefit for patients with SCLC.

**Supplementary Information:**

The online version contains supplementary material available at 10.1186/s12935-021-02357-1.

## Introduction

Small cell lung cancer (SCLC) is the most malignant subtype of lung cancer, accounting for 13–15% of all lung cancer cases [1]. SCLC is aggressive, features rapid growth, is characterized by early metastases, and is the sixth most-common cause of cancer-related death [2]. These factors have led to limited improvements in the typical life span of patients with SCLC treated by standard therapy in the past few decades. The median overall survival (OS) of patients with SCLC has stalled at fewer than 10 months and the disease features a dismal 5-year survival rate of 5% [3]. Currently, platinum-based chemotherapy remains the first-line treatment for SCLC; however, the challenge of drug resistance has not been well addressed [4]. Considering the high mortality rate of SCLC patients, there is an urgent need for new biomarkers to facilitate improvements in methods of diagnosis, treatment, and prognosis. Immune responses play a pivotal role in tumor development and progression, and they may also influence the survival of patients with SCLC [5–7]. Immune cells mediate tumor immune responses and are closely associated with tumor growth, invasion, and metastasis [8, 9]. Notably, recent research found immune cell infiltration to be a key determinant of prognosis for patients with SCLC. Furthermore, the different immune cell profiles in the tumor microenvironment often affect the survival of patients with SCLC [10]. In addition, emerging immunotherapy strategies with immune checkpoint inhibitors have achieved promising progress as treatments for various malignancies, and such methods show significant promise as treatments for SCLC patients [11, 12]. Therefore, the influence of immune-related factors on the development and progression of SCLC warrants investigation.

With considerable recent advancements in transcriptome sequencing technology, the crucial role of long non-coding RNAs (lncRNAs) in tumorigenesis and progression has been elucidated [13]. LncRNAs are a subtype of non-coding RNA transcript ranging from 200 nucleotides to 100 kilobases in length [14, 15]. LncRNAs contribute to malignant tumor phenotypes by regulating genomic and transcriptomic alterations and affecting the tumor immune microenvironment [16]. Importantly, lncRNAs actively regulate expression of genes related to immune responses and activation, increasing the heterogeneity of the tumor immune microenvironment by encouraging infiltration of different immune cells [17]. Several signatures based on tumor immune infiltration have been shown to be reliable and promising tools for the diagnosis, treatment, and prognostication of various tumors, and lncRNAs have been included in some of these signatures [18–20]. Immune-related lncRNA (irlncRNA)-based signatures have been found to be useful prognostic tools for several types of tumors, including pancreatic cancer, breast cancer, and hepatocellular carcinoma [21–23]. However, few attempts have been made to explore the value of irlncRNAs for prognosis prediction in SCLC, and the clinical utility of an irlncRNA-based signature in patients with SCLC has not been well established.

This study includes the first published irlncRNA expression profile for patients with SCLC. In addition, an eight-irlncRNA signature was used to stratify the adjuvant chemotherapeutic response and prognostic risk of a training cohort of SCLC patients, and the prognostic value of this signature was well-validated in a validation cohort. We also explored the relationship between the irlncRNAs in the signature and tumor immunity. Thus, our eight-irlncRNA signature may serve as a promising prognostic predictor for SCLC and may guide the clinical application of chemotherapy and immunotherapy for SCLC patients.

## Materials and methods

### Patients and immune gene sets

We collected data for 227 patients with SCLC for this study, including 79 samples from a publically available database (GSE60052) at the Gene Expression Omnibus (GEO) (http://www.ncbi.nlm.nih.gov/geo), and 148 cases with formalin-fixed and paraffin-embedded (FFPE) tissues who underwent surgery at the Chinese Academy Medical Sciences Cancer Hospital from 2009 to 2018. All cases were of Asian descent. Among the 148 cases, 128 patients had received adjuvant chemotherapy. SCLC was pathologically re-confirmed for all patients. The clinical features of enrolled patients were displayed in Table 1. The starting point for OS and relapse-free survival (RFS) was defined as the day of surgery; the endpoint was the day of death or the last follow-up and the date of relapse metastasis, or the last follow-up. This research was approved by the Institutional Review Board of The Chinese Academy Medical Sciences Cancer Hospital.

**Table 1:** Clinical characteristics of the patients from different cohorts

Characteristics	Training cohort (*N* = 48)	Validation cohort (*N* = 148)
Age, year		
< 60	27 (56.25%)	79 (53.38%)
≥ 60	21 (43.75%)	69 (46.62%)
Sex		
Male	43 (89.58%)	116 (78.38%)
Female	5 (10.42%)	32 (21.62%)
Smoking history		
Yes	33 (68.75%)	92 (62.16%)
No	15 (31.25%)	56 (37.84%)
SCLC staging		
I	8 (16.67%)	54 (36.49%)
II	8 (16.67%)	48 (32.43%)
III	31 (62.50%)	46 (31.08%)
IV	1 (2.08%)	0 (0.00%)
OS state		
Alive	25 (52.08%)	68 (45.95%)
Death	23 (47.92%)	80 (54.05%)

*SCLC* small cell lung cancer; *OS* overall survival

### Identification of immune-related lncRNAs

Firstly, we obtained the lncRNA profile, based on previous literature, with gene expression microarray data [24]. The GSE60052 data was first log2-transformed and quantile-normalized, after which we applied the annotation file (GPL11154) with gene code v36 IDs. Based on this mapping procedure, the lncRNA profiles of 2942 lncRNAs were determined. The list of immune genes was based on the NanoString nCounter PanCancer Immune Profiling Panel (LBL-10043-08), and 764 immune genes were identified. LncRNAs and immune genes with low expression levels (where half or more than half of values were 0) were filtered out. Next, a Pearson correlation analysis was conducted between the final 607 immune genes and 1202 lncRNAs. During this analysis, only lncRNAs with |R| >  0.6 and *P * <  0.001 were identified as irlncRNAs. Finally, 316 irlncRNAs were included in this investigation.

### Functional analysis

We determined which genes were associated with irlncRNAs using the Multi Experiment Matrix website (http://biit.cs.ut.ee/mem/). The selected genes were uploaded to the DAVID 6.8 (http://david.abcc.ncifcrf.gov/home.jsp) website and subjected to Gene Ontology (GO) and Kyoto Encyclopedia of Genes and Genomes (KEGG) analyses. In addition, Gene set enrichment analysis (GESA, http://www.broadinstitute.org/gesa/index.jsp) was used to explore the related signaling pathways between high- and low-risk groups. Finally, the GSVA R package was applied to run the Gene Set Variation Analysis (GSVA) function in R software (version 3.5.1).

### RNA isolation and qPCR analysis

We extracted the total RNA of FFPE surgical specimens using the Ambion RecoverAll Total Nucleic Acid Isolation Kit for FFPE (ThermoFisher, Waltham, MA, USA) in December 2020. The target irlncRNA expression levels were evaluated by qPR-PCR analysis of a 10-μL volume system in triplicate on a 7900HT Fast Real-Time PCR system (Applied Biosystems, Carlsbad, USA, Indianapolis, IN) using the SYBR Green Master Mix method (Invitrogen). The 2^−ΔΔCt^ method was selected to assess the expression of all candidates. All primer sequences of selective irlncRNAs are included in Additional file 2: Table S1. The related expression data was presented in Additional file 2: Table S2.

### Establishment of risk signature and statistical analysis

To construct a reliable risk signature, we first used a univariate Cox regression model to identify irlncRNAs with prognostic potential. We further filtered out the candidates that were significantly associated with survival to generate a risk signature based on the least absolute shrinkage and selection operator (LASSO) model. The risk score was calculated using the expression levels of eight selected irlncRNAs and their corresponding coefficients. Risk scores were calculated for all patients in this study. Pearson correlation analysis was applied to determine the correlations between the risk score and classical immune checkpoints. A multivariate Cox proportional hazards regression analysis was carried out to determine whether the signature could independently predict prognosis for SCLC. The OS and RFS of patients in different risk groups were assessed by Kaplan–Meier survival analysis. ROC curves were evaluated to assess the predictive capacity of various clinical parameters, TNM stage, and risk score for OS using R software (version 3.5.1). All image production and data analyses in this study were performed using SPSS 25.0 and R software (version 3.5.1). *P* values less than 0.05 were considered significant, and all tests were two-tailed.

### Results

#### Identification of prognostic irlncRNAs from the training cohort

**Fig. 1:**
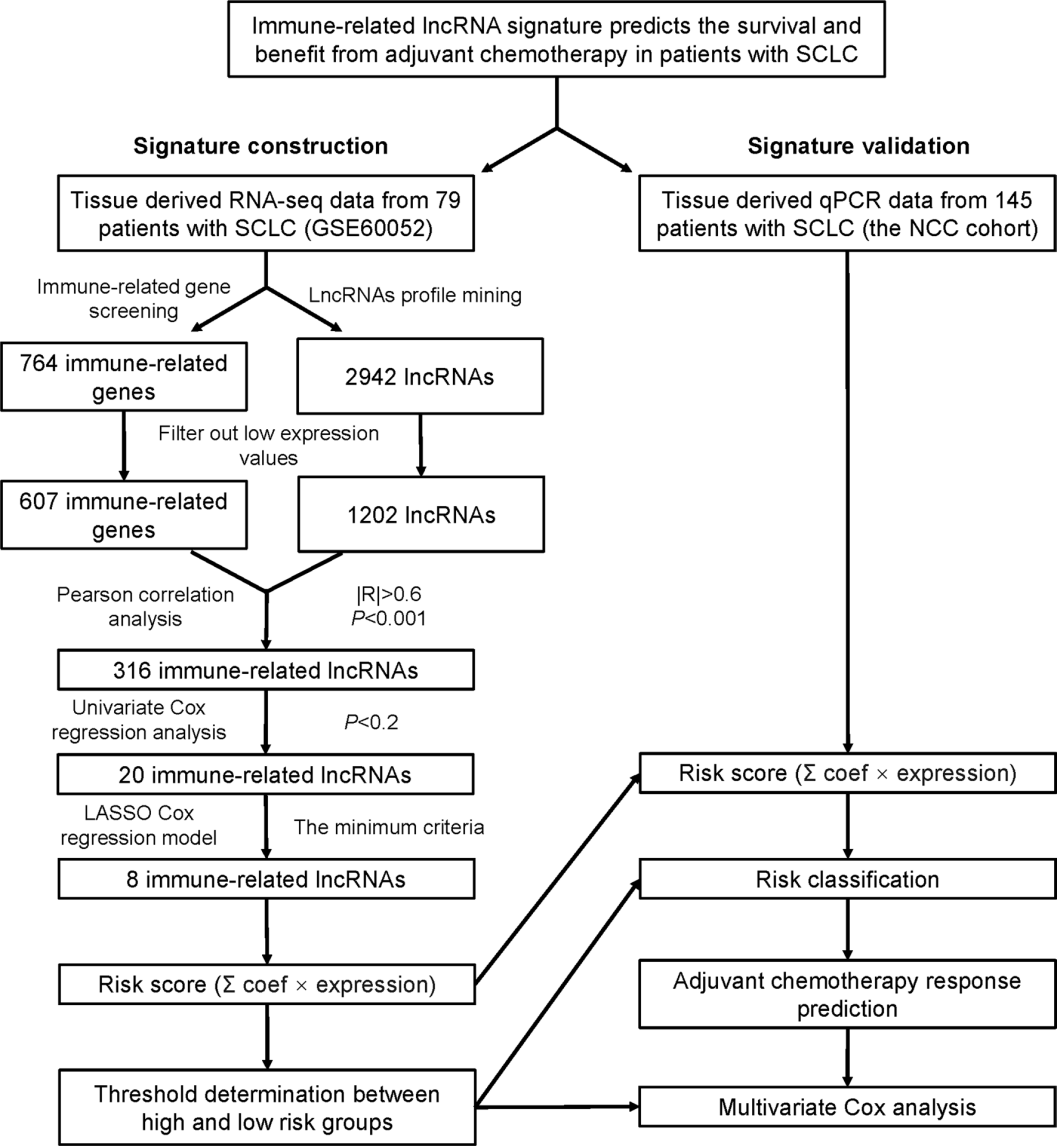
Flow chart of this study

**Fig. 2:**
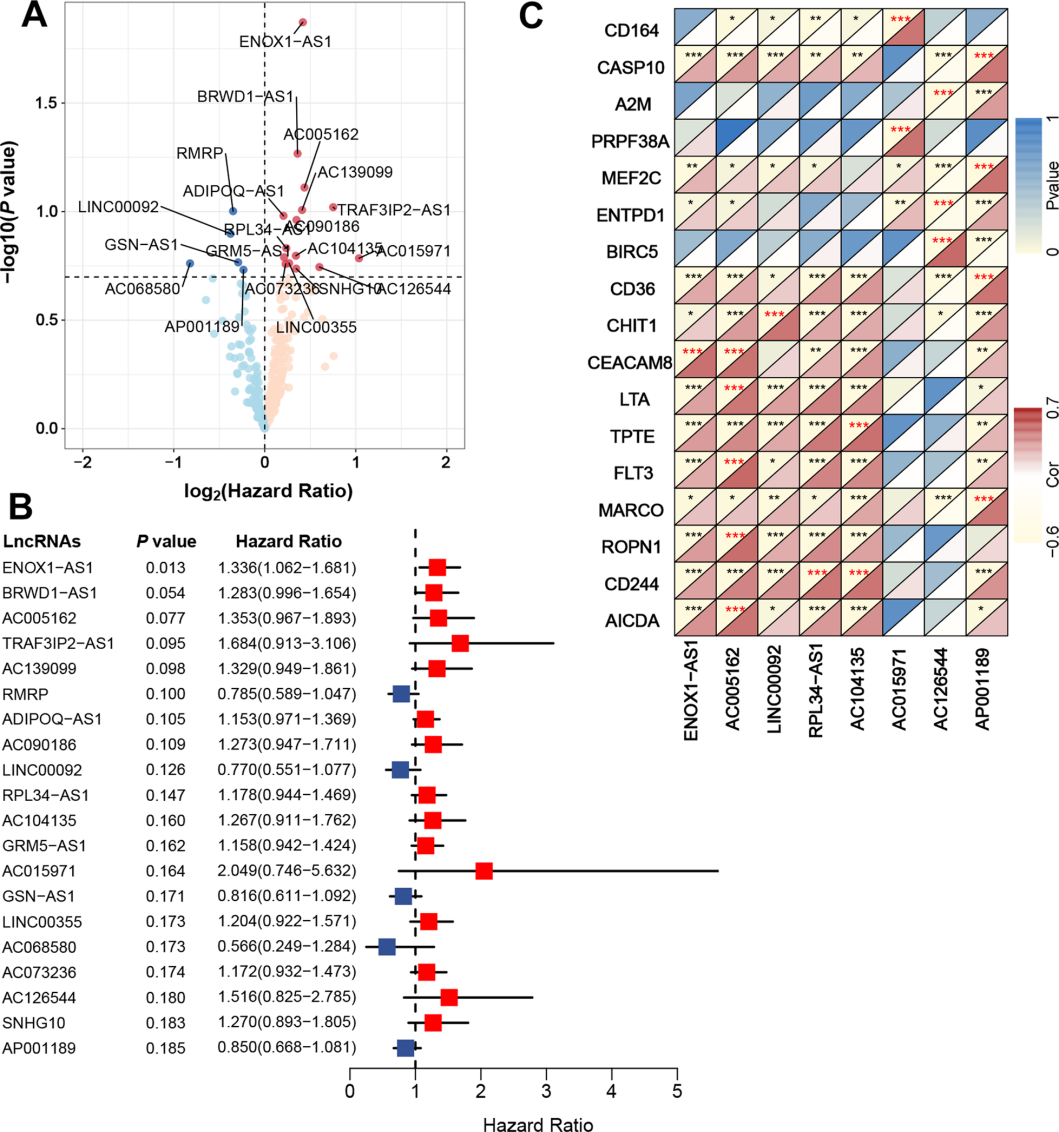
Filter out the most significant prognostic irlncRNAs in small cell lung cancer. **a** Univariate cox regression analysis filtered out 20 significant prognostic irlncRNAs. **b** Forest plot of the association between irlncRNAs and prognosis in SCLC. **c** Correlation between irlncRNAs and immune genes

The flow chart of this study is presented in Fig. 1. Firstly, 764 immune genes and 2942 lncRNAs were identified in 79 cases from GSE60052. Then, we sought to determine the prognostic value of irlncRNAs in patients with SCLC. We mapped the gene code IDs to the annotation files in GSE60052, including 79 SCLC samples. To ensure that our analysis had clinical significance, only immune genes and lncRNAs with high expression levels were included. After filtering out the low-expression candidates, only 607 immune genes and 1202 lncRNAs were selected for further exploration. Pearson correlation analysis (|R| > 0.6 and *P*  < 0.0001) was conducted between these 607 immune genes and 1202 lncRNAs, and 316 irlncRNAs were selected in this procedure. Next, we performed univariate Cox regression analysis on survival data from 48 patients in GSE65002, and 20 prognostic irlncRNAs were selected (Fig. 2A, B, *P*  < 0.2). For the next step, we performed LASSO regression analysis on these 20 irlncRNAs to identify the most promising candidates, and the minimum criteria were chosen (Additional file 1: Figure S1). Finally, eight irlncRNAs were selected for the irlncRNA signature: ENOX1-AS1, AC005162, LINC00092, RPL34-AS1, AC104135, AC015971, AC126544, and AP001189. The immune genes associated with the 8 selected irlncRNAs are displayed in Fig. 2C.

#### Construction of an irlncRNA signature for SCLC

**Fig. 3:**
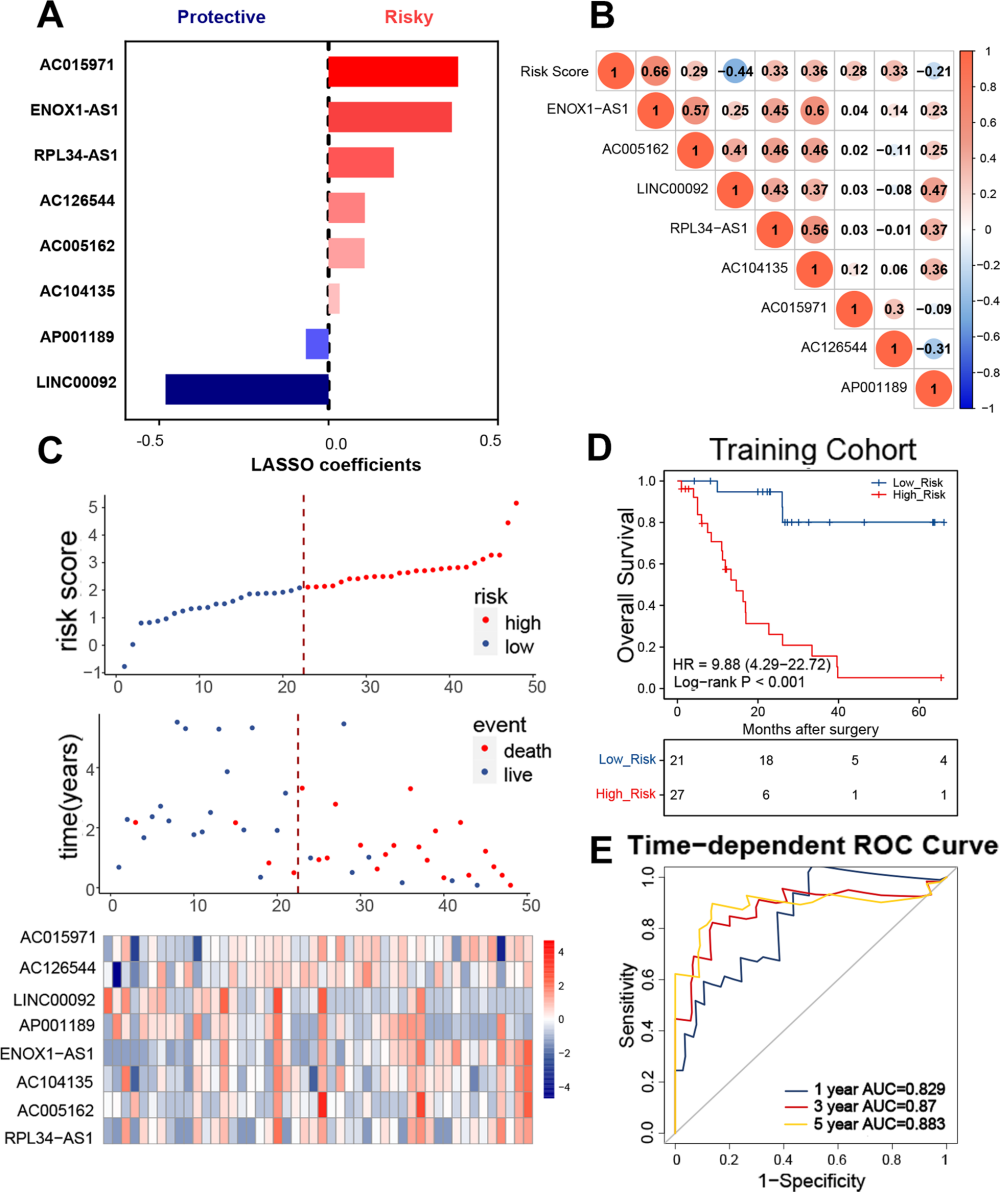
The irlncRNA signature distribution and survival of patients in the training cohort. **a** LASSO Cox coefficient profiles of the selected prognostic irlncRNAs. **b** Correlation between the expression of selected irlncRNAs and risk score. **c** Risk score distribution with patient survival status in the training cohort, with red color indicating that patients have died and blue color indicating survival. Expression distribution of the eight irlncRNAs in the training cohort, with red color indicating higher expression and blue indicating lower expression. **d** Kaplan–Meier curves of OS in 48 patients from the training cohort based on risk score. **e** ROC analysis of the irlncRNA signature for prediction of survival at 1, 3, and 5 years in the training cohort

The eight irlncRNAs and their corresponding coefficients were combined to establish a molecular risk score model for patients with SCLC, including two protective factors and six risk factors, as follows: risk score  =  (0.3647  ×  ENOX1-AS1 expression)  +  (0.1062 × AC005162 expression)  +  (0.1935 × RPL34-AS1 expression)  +  (0.0329 × AC104135 expression)  +  (0.3833  ×  AC015971 expression)  +  (0.1074 × AC126544 expression) − (0.4814  ×  LINC00092 expression) − (0.0665  ×  AP001189 expression) (Fig. 3A). The relationship between the irlncRNA signature and the corresponding risk score is shown in Fig. 3B. Based on the irlncRNA signature, all patients in the training cohort were assigned individual risk scores. Then, patients were classified into low- and high-risk groups according to the optimal cut-off value (Fig. 3C). The Kaplan–Meier curves demonstrate that high-risk patients had shorter OS than their low-risk counterparts (Fig. 3D). We also evaluated the accuracy of the irlncRNA signature for predicting the OS of SCLC patients by performing time-dependent ROC curve analysis. The AUC of the training cohort was 0.829 at 1-year, 0.87 at 3-year, and 0.883 at 5-years. These results suggest that the irlncRNA signature generated here is an excellent predictor of OS for SCLC patients.

#### Validation of the irlncRNA signature in SCLC

**Fig. 4:**
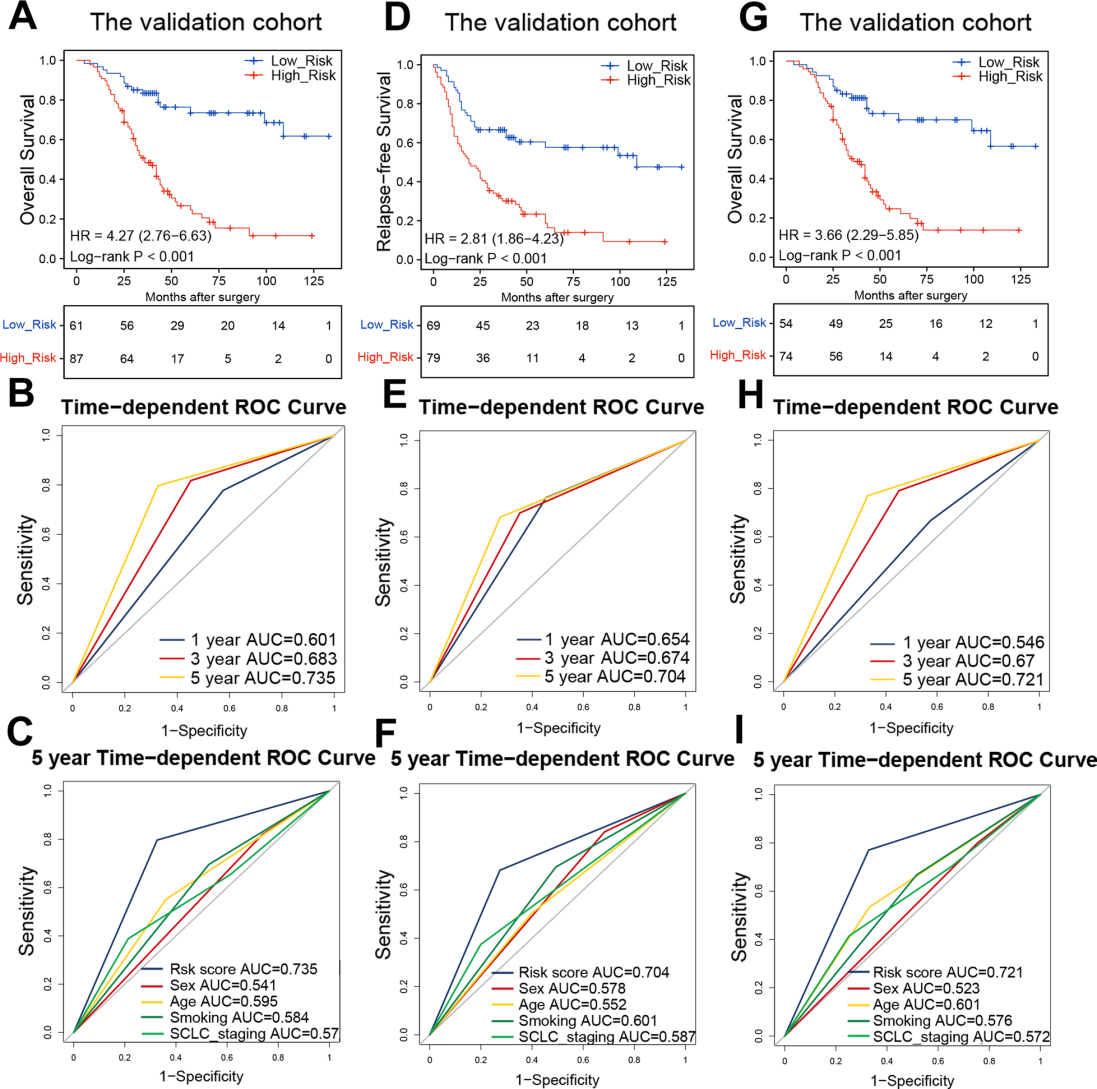
Validating the irlncRNA signature in the validation cohort with qPCR data. **a** Kaplan–Meier curves of OS for 148 patients of the validation cohort based on risk score. **b** ROC analysis of risk score for prediction of survival at 1, 3, and 5 years for the validation cohort. **c** ROC analysis of risk score and different clinical parameters for OS for the validation cohort. **d** Kaplan–Meier curves of RFS for 148 patients of the validation cohort based on risk score. **e** ROC analysis of risk score for prediction of RFS at 1, 3, and 5 years for the independent cohort. **f** ROC analysis of risk score and different clinical parameters for RFS for the independent cohort. **g** Kaplan–Meier curves of OS for the ACT subgroup of the validation cohort based on risk score. **h** ROC analysis of risk score for prediction of OS at 1, 3, and 5 years for the ACT subgroup of the validation cohort. **i** ROC analysis of risk score and different clinical parameters for RFS for the ACT subgroup of the validation cohort

To examine whether our signature has potential clinical applications, we further validated it in a validation cohort including 148 patients with SCLC. Based on the qPCR analysis, all patients had measurable expression levels of the eight selected irlncRNAs. Similar to the analysis described above, we calculated risk scores for all patients and classified them into low- and high-risk groups. Again, low-risk patients demonstrated better OS than their high-risk counterparts (Fig. 4A). To validate the robustness and optimality of the irlncRNA signature, we conducted ROC curve analysis for 1-year, 3-year and 5-year OS, and all AUCs exceeded 0.6 (Fig. 4B). We also compared the 5-year ROC curves between our irlncRNA signature and other important clinical parameters. The irlncRNA signature exhibited excellent discriminatory capacity, with a 5-year AUC as high as 0.735 (Fig. 4C). Furthermore, we tested the predictive efficacy of our signature for the RFS of patients with SCLC. Low-risk patients had RFS better than that of high-risk patients (Fig. 4D). The AUCs of the irlncRNA signature for predicting 1-year, 3-year and 5-year RFS were 0.654, 0.674, and 0.704, respectively (Fig. 4E). The irlncRNA signature was also superior to other critical clinical features as a predictor of 5-year RFS for SCLC patients (Fig. 4F).

**Fig. 5:**
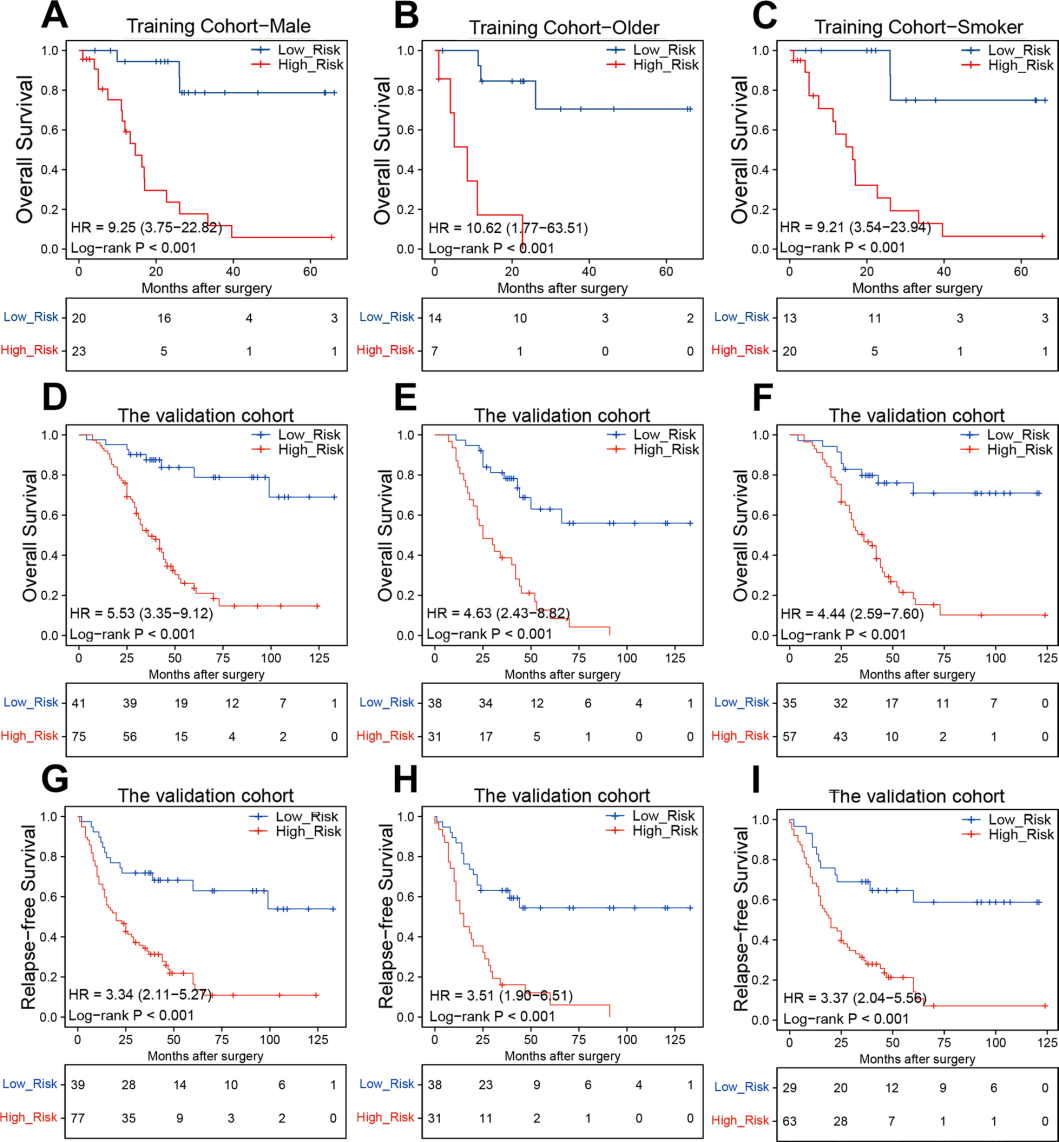
Validation of the OS and RFS predictive performance of the risk score across clinical subgroups. **a** Kaplan–Meier curves of OS for males from the training cohort. **b** Kaplan–Meier curves of OS for older patients from the training cohort. **c** Kaplan–Meier curves of OS for smokers from the training cohort. **d** Kaplan–Meier curves of OS for males from the validation cohort. **e** Kaplan–Meier curves of OS for older patients from the validation cohort. **f** Kaplan–Meier curves of OS for smokers from the validation cohort. **g** Kaplan–Meier curves of RFS for males from the validation cohort. **h** Kaplan–Meier curves of RFS for older patients from the validation cohort. **i** Kaplan–Meier curves of RFS for smokers from the validation cohort

Since adjuvant chemotherapy (ACT) is the preferred approach for SCLC treatment, we investigated the relationship between our irlncRNA signature and OS in patients who received ACT within the validation cohort described above. In the ACT subgroup, high-risk cases also suffered unfavorable prognoses (Fig. 4G). The 1-year, 3-year, and 5-year OS ROC curves are illustrated in Fig. 4H. Compared to other clinical characteristics, the irlncRNA signature showed the highest AUC for 5-year OS prediction in the ACT subgroup (Fig. 4I). We further validated the signature in subgroups of patients with important clinical characteristics from the training and validation cohorts. Notably, high-risk patients exhibited poorer OS in the clinical parameter subgroups in the training cohort, including males, older patients, and smokers (Fig. 5A–C). The same results were observed in the validation cohort; high-risk cases showed inferior OS and RFS in the subgroups of males, older patients, and smokers (Fig. 5D–I). Taken together, these results demonstrate that our irlncRNA signature is a reliable and effective predictor of OS for SCLC patients.

#### The irlncRNA signature is an independent prognostic factor for SCLC patients

**Fig. 6:**
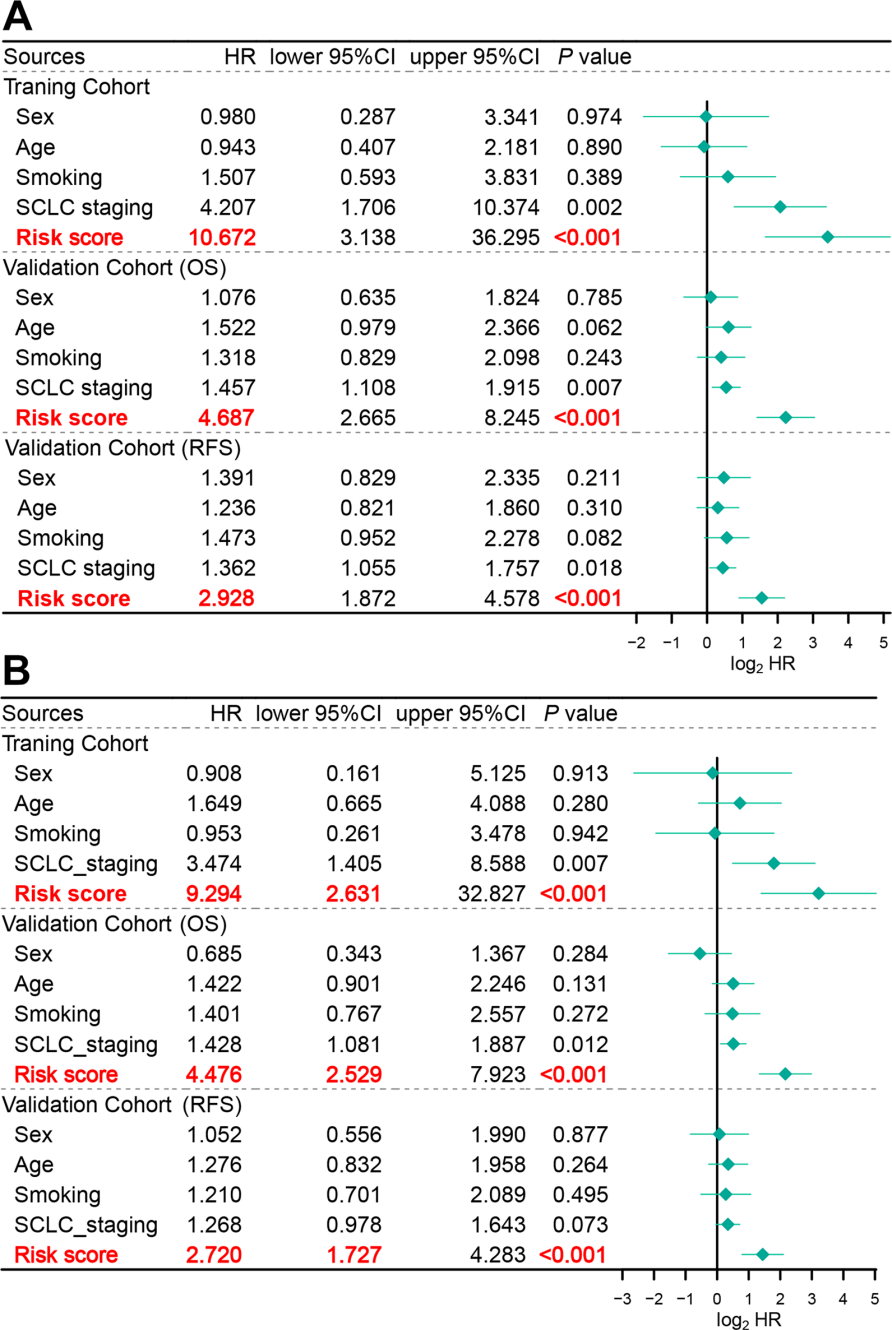
Cox regression analyses of the irlncRNA signature in the training and validation cohorts. **a** Univariate Cox regression analyses of the risk score and clinical parameters. **b** Multivariate Cox regression analyses of the risk score and clinical parameters

To determine whether the irlncRNA signature is an independent prognostic factor for SCLC patients, we conducted univariate and multivariate Cox regression analyses using the training and validation cohorts. In comparison with other clinical parameters, the risk score was most significantly related to the prognosis of SCLC for the training and validation sets (Fig. 6A). Various clinical parameters, including sex, age, smoking, and SCLC staging, were incorporated in the multivariate Cox regression analysis. Importantly, the risk score was shown to function as an independent predictor of OS and RFS for patients with SCLC (Fig. 6B). Collectively, these results demonstrate that the irlncRNA signature can predict risk and effectively stratify patients with SCLC.

#### Functional analysis of the irlncRNA signature

**Fig. 7:**
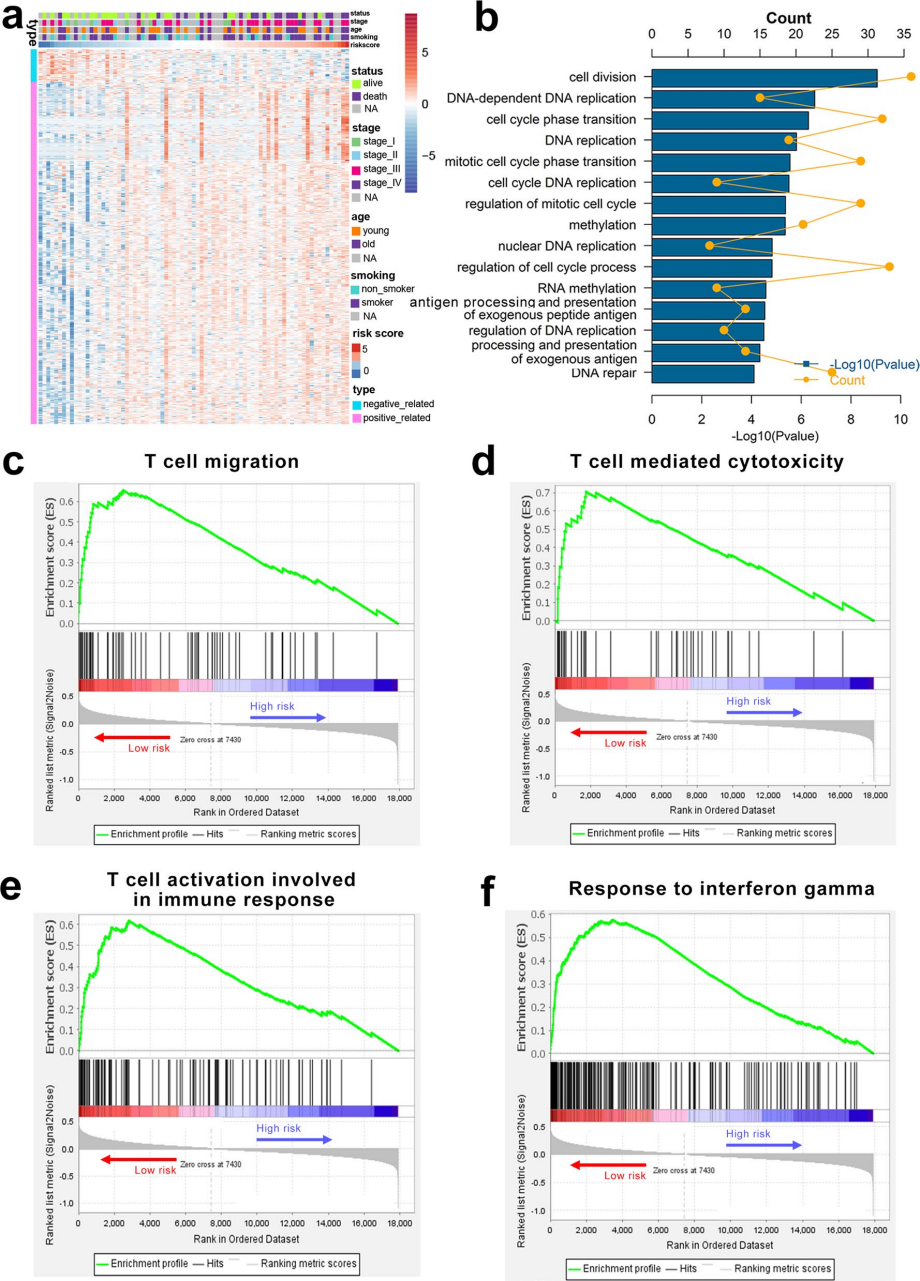
Functional analysis of the irlncRNA signature in the training cohort. **a** Details of the risk score and the most relevant genes. **b** Gene enrichment with the GO terms of the selected genes. **c**–**f** Gene set enrichment analysis indicated a significantly activated immune phenotype in the low-risk cases

GO analysis was used to investigate the biological significance of the irlncRNA signature. We first identified 527 genes with Pearson |R| >  0.35 (481 positively related and 46 negatively related) that were strongly related to the irlncRNA signature. A heatmap of these genes and the distribution of clinical parameters for all patients in the training cohort are illustrated in Fig. 7A. The GO analysis indicated that the irlncRNA signature was related to several cell proliferation-related pathways, including cell division and various DNA replication pathways. These findings are consistent with the malignant biological characteristics of SCLC, including rapid proliferation (Fig. 7B). We also found that the risk score was associated with antigen processing and presentation pathways, suggesting that the irlncRNAs in the signature could be associated with T cell function (Fig. 7B). Gene set enrichment analysis (GSEA) was performed to further validate the relationship between the irlncRNA signature and immune activity. The GSEA results showed that the risk level of low-risk patients was positively related to T cell migration (*P*  <  0.001), T cell mediated cytotoxicity (*P*  <  0.001), T cell activation involved in immune responses (*P*  =  0.016), and response to INF-γ (*P*  =  0.033), suggesting an activated immune phenotype.

#### Relationship between the irlncRNA signature and the immune landscape

**Fig. 8:**
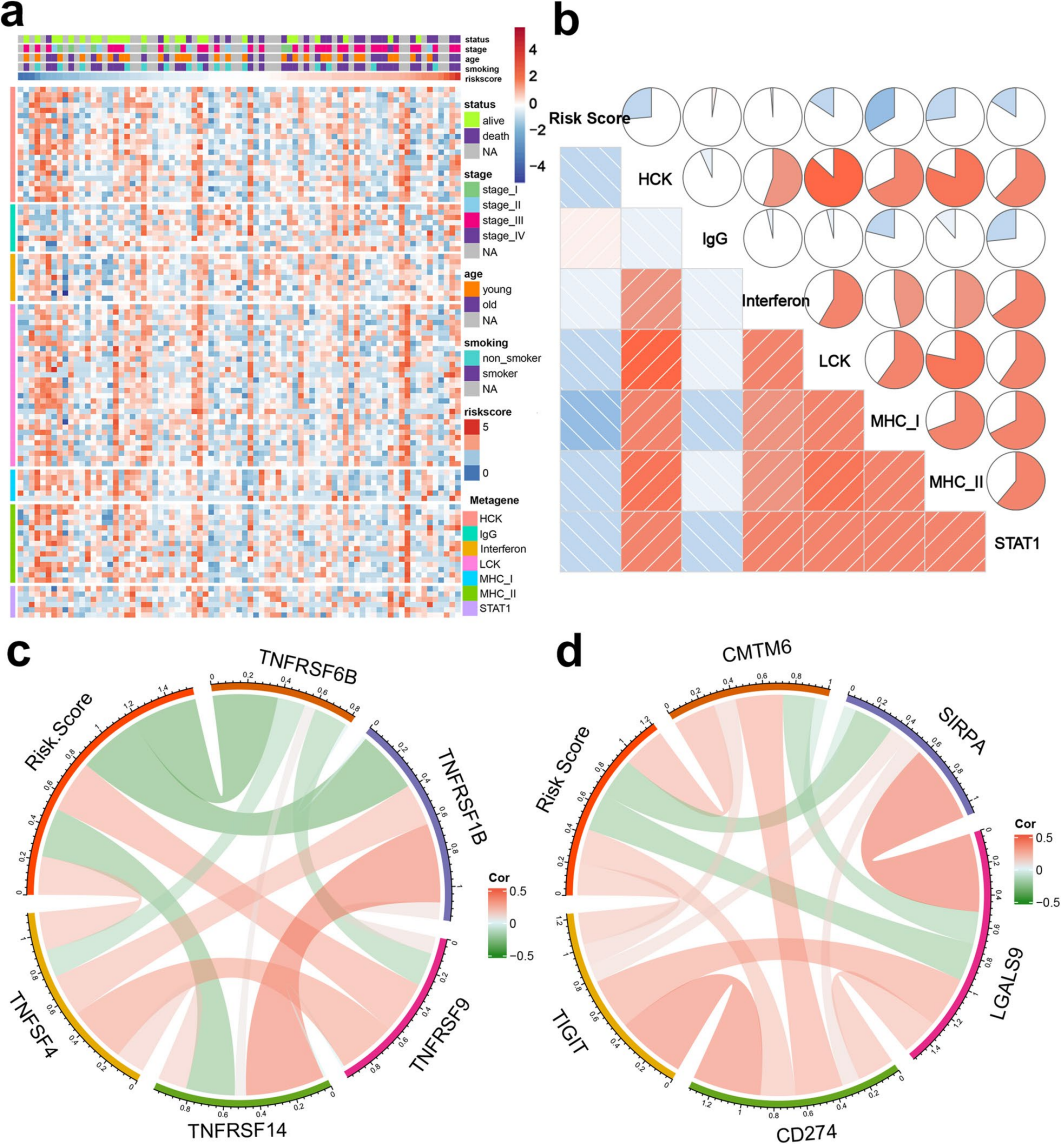
Relationship between risk scores and inflammatory metagenes and immune checkpoints. **a**, **b** Metagene heatmap and corrgram for the training cohort. **c**, **d** Correlation between risk score and immune checkpoint expression

To comprehensively assess the relationship between the risk score and the immune landscape, seven clusters of inflammatory and immune response metagenes (HCK, interferon, LCK, MHC-I, MHC-II and STATA) were chosen for further exploration [25, 26]. As displayed in Fig. 8A, the risk score was negatively correlated with most clusters, including HCK, LCK, and MHC-II. These seven metagene clusters were subjected to GSVA for further validation. Corrgrams were generated based on the Pearson r value between risk score and the seven metagenes (Fig. 8B). Here, the risk score was negatively correlated with LCK, MHC-I, MHC-II, and STAT1. Thus, low-risk patients demonstrated activated macrophages and T cell signaling transduction.

Considering the pivotal roles that immune checkpoints play in tumor immunity, we investigated the correlation between the risk score and the expression levels of several essential immune checkpoints. The risk score was positively related to TNFSF4, TNFRSF9, CMTM6, TIGIT, and CD274 (Fig. 8C, D). TNFSF4 and TNFRSF9 are critical members of the TNF family, and immunotherapies targeting TNFSF4 have achieved promising results in patients with some malignancies [27]. In addition, CTMT6 and TIGIT have been identified as novel immune checkpoints that can be targeted by tumor immunotherapies [28, 29]. In summary, these results indicate that high-risk patients are likely to benefit from novel immunotherapeutic treatments targeting the checkpoints listed above.

## Discussion

SCLC is considered the most fatal type of lung cancer, with limited treatment options and a dismal prognosis. The prognosis and response to treatment vary widely among SCLC patients with similar clinical characteristics because SCLC is a highly heterogeneous tumor with significant genetic diversity [30]. Therefore, we must identify novel molecular biomarkers (different from traditional clinical risk parameters) for predicting treatment response and prognosis for patients with SCLC. In the past decade, several mRNA-based and miRNA-based molecular methods have been proposed to predict the prognosis of patients with SCLC [31, 32]. Recently, with the advancement of high-sequencing technology, lncRNA expression dysregulation was identified in various malignancies. These findings underscore the critical function of lncRNAs in tumorigenesis and development [33, 34]. Furthermore, lncRNAs appear to be involved in genomic and transcriptomic regulation, and they are known to affect tumor immunity [16]. Other studies have found that irlncRNAs may represent promising therapeutic targets and predictive biomarkers for clinical management and precision therapy for patients with different types of tumors [35, 36]; however, there has been little exploration of the prognostic significance of irlncRNAs in SCLC patients.

Therefore, in the present study, we constructed an irlncRNA signature and explored its prognostic value for SCLC, as well as its value as a tool for predicting the response of patients with SCLC to ACT. The signature effectively stratified various risk factors for accurate prognostication of patients with SCLC, and it was further validated in the validation cohort, showing that it could independently predict the OS and RFS of SCLC patients. The high-risk patients suffered a worse prognosis and benefited little from adjuvant chemotherapy in comparison with their low-risk counterparts. Notably, compared with various well-recognized clinicopathologic traits, our signature had better predictive performance for ACT response and prognosis for patients with SCLC. We also explored the relationship between the signature and tumor immunity, which may provide targets for immunotherapies for SCLC patients.

Eight irlncRNAs (ENOX1-AS1, AC005162, LINC00092, RPL34-AS1, AC104135, AC015971, AC126544, and AP001189) were included in the risk classifier to predict prognosis for patients with SCLC. AC005162 promotes breast cancer cell growth, while low expression of AC005162 is associated with a better prognosis for breast cancer patients [37]. LINC00092 alters glycolysis to support the function of cancer-associated fibroblasts, which accelerates ovarian cancer tumor progression and metastasis [38]. Lower LINC00092 expression was shown to be related to favorable prognosis for patients with colon adenocarcinoma or breast cancer, and a poorer prognosis for patients with lung adenocarcinoma [39–41]. RPL34-AS1 was found to be down-regulated in various malignancies, including colorectal, gastric, and esophageal cancers [42]. In esophageal cancer, RPL34-AS1 inhibits tumor proliferation, migration, and invasion by regulating the expression of RPL34, which serves as a tumor suppressor to inhibit tumorigenesis and development [42]. AC104135 was found to be highly expressed in breast cancer and was shown to be a risk factor for breast cancer in a Chinese population [43]. High expression of AP001189 was reported to be closely associated with a better prognosis for colon cancer [44]. Few studies have explored the roles of ENOX1-AS1, AC015971, and AC126544; additional research is needed to uncover their tumor-related functions. Additionally, the roles of these eight lncRNAs in SCLC development are poorly understood, and further relevant studies are needed to explore their functions.

We also explored potential risk signature mechanisms. It was found that the genes associated with our signature were associated with cell division and multiple DNA replication pathways, in accordance with the rapid proliferation features of SCLC. The selected irlncRNAs appear to be involved in immune cell responses. Patients in the high-risk and low-risk groups featured different immune statuses. The risk level of the low-risk patients was positively related to T cell migration, T cell-mediated cytotoxicity, T cell activation involved in immune responses, and response to INF-γ, demonstrating a different immune phenotype activation pattern in comparison with that of the high-risk group. In addition, the irlncRNA expression levels of low-risk patients suggesting activated macrophages and T cell signaling transduction. The risk score was positively correlated with several novel immune checkpoints, including TNFSF4, TNFRSF9, CMTM6, TIGIT, and CD274. TNFSF4 and TNFRSF9 are critical members of the TNF family. They contribute to co-stimulatory or co-inhibitory signals of T cell immune responses, and immunotherapies targeting TNFSF4 in some malignancies are promising [27]. CMTM6 is a PD-L1 protein regulator that helps maintain the expression of PD-L1 while regulating tumor immunity [45]. TIGIT is an inhibitory receptor in CD8  +  T cells that limits their function and promotes T cell exhaustion [46]. Both CMTM6 and TIGIT are considered to be promising therapeutic targets [45, 46]. The irlncRNA signature reported in this study may facilitate clinical application of novel immunotherapies for patients with SCLC in the future.

We created the first comprehensive lncRNA profile of SCLC and confirmed that lncRNAs are useful for predicting the prognosis of SCLC patients. Additional experiments are urgently needed to explore the functions and molecular mechanisms of lncRNAs in SCLC. Additionally, considering the pivotal role of immunotherapy, we proposed a potential novel immune-related therapeutic approach and prognostic target for SCLC. Our molecular signature was well validated in tissue specimens from the validation cohort, suggesting that our classifier is reliable and clinically useful.

Despite these promising preliminary findings, several limitations warrant consideration. Firstly, we used lncRNA profiles based on RNA-seq data from the GEO. It is likely that most, but not all, of the potential lncRNAs were included in the analysis. Future investigations should explore the biological functions of these lncRNAs. Second, the small sample size of the GEO database training cohort limited the molecular modeling process; more cohorts with larger sample sizes are needed in the future. Lastly, this was retrospective research featuring validation of FFPE specimens, and prospective samples are needed for in-depth validation.

In conclusion, we have comprehensively revealed the expression profile of irlncRNAs in SCLC and constructed an eight-irlncRNA classifier to predict prognostic risk for patients with SCLC. In the clinic, this classifier may enable effective screening of high-risk SCLC patients. For high-risk patients, early positive interventions, including timely receipt of postoperative adjuvant therapy and clinical management, should be provided. Furthermore, the eight-irlncRNA signature can be used to identify patients who are likely to be resistant to traditional adjuvant chemotherapy, which may prompt them to try emerging immunotherapies and enroll in clinical trials. The irlncRNA signature reported here could be useful for clinical management of SCLC patients and as a tool for determining whether patients are likely to benefit from chemotherapy.

## Supplementary Information

**Additional file 1: Figure S1.** **a**, **b** 100-fold cross-validation for tuning parameter selection in a LASSO Cox model.

**Additional file 2: Table S1.** Primer sequences used in the validation cohort for qPCR. **Table S2.** The expression data of enrolled immune-related lncRNAs in SCLC.

## Data Availability

Data and materials related to this work are available upon request.

## Abbreviations

SCLC: Small cell lung cancer
OS: Overall survival
lncRNA: Long non-coding RNA
irlncRNAs: Immune-related lncRNAs
FFPE: Formalin-fixed and paraffin-embedded
RFS: Relapse-free survival
GO: Gene ontology
KEGG: Kyoto Encyclopedia of Genes and Genomes
GSEA: Gene set enrichment analysis
GSVA: Gene set variation analysis
LASSO: Least absolute shrinkage and selection operator
