# Supplementary figures and images for: An immune-related lncRNA signature predicts prognosis and adjuvant chemotherapeutic response in patients with small-cell lung cancer
Source: Cancer Cell Int. 2021 Dec 20;21:691. doi: 10.1186/s12935-021-02357-1 (PMC8691030; doi:10.1186/s12935-021-02357-1)

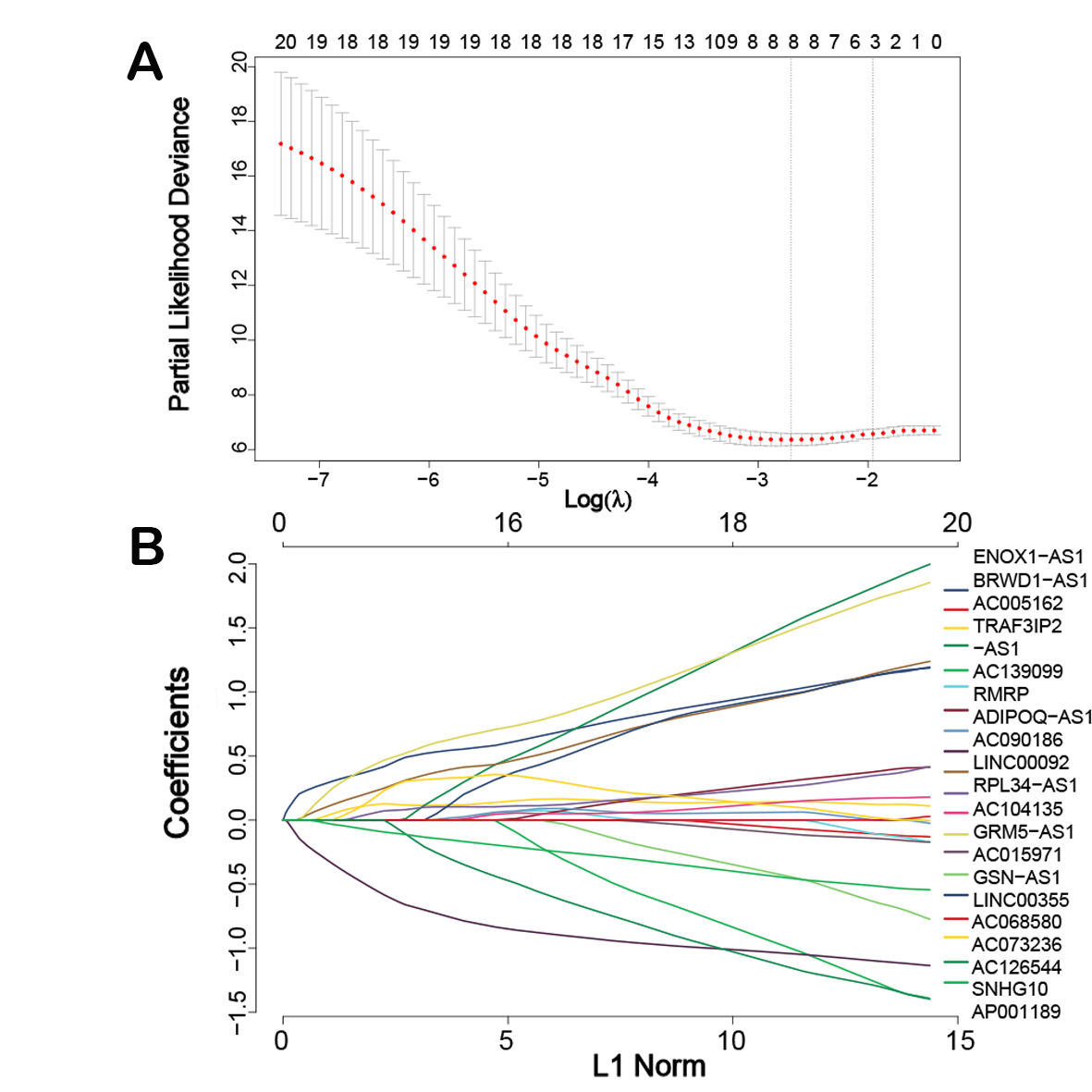

Supplement: Supplementary file 1 — Additional file 1: Figure S1. a, b 100-fold cross-validation for tuning parameter selection in a LASSO Cox model. [file 12935_2021_2357_MOESM1_ESM.tif]
