# Supplementary material for: An immune-related lncRNA signature predicts prognosis and adjuvant chemotherapeutic response in patients with small-cell lung cancer
Source: Cancer Cell Int. 2021 Dec 20;21:691. doi: 10.1186/s12935-021-02357-1 (PMC8691030; doi:10.1186/s12935-021-02357-1)
Supplement: Supplementary file 2 — Additional file 2: Table S1. Primer sequences used in the validation cohort for qPCR. Table S2. The expression data of enrolled immune-related lncRNAs in SCLC. [file 12935_2021_2357_MOESM2_ESM.docx]

**Supplementary Table S1**. Primer sequences used in the validation cohort for qPCR.

| LncRNAs | Forward Primer | Reverse Primer |
| --- | --- | --- |
| ENOX1-AS1 | 5'- TGCACTGCCATGTGTTTTCA -3' | 5'- TCCGCCTCTGAGGACACTATAA -3' |
| AC005162 | 5'- AGGATCCCATCCTTTTCCATAAA -3' | 5'- ACCCTATGACCAGCCTCTGA -3' |
| LINC00092 | 5'- TGGGTGAATTGTCGGAAGCA -3' | 5'- TTGGCCGTTAAAACCCCAGA -3' |
| RPL34-AS1 | 5'- AAAGCCGCAAAGAGCAAAGG -3' | 5'- GAGTGGTAGCGATGGAGAGC -3' |
| AC104135 | 5'- AAGGACCTGGCTCCGAAATG -3' | 5'- CTATCGCTGTCCAGCCTCAC -3' |
| AC015971 | 5'- AGCTACCTCTTCCACCCGAT -3' | 5'- GTGAAGCCAAAGGTCAGGGA -3' |
| AC126544 | 5'- AGGGTATGGTTGAACGTCTCTG -3' | 5'- TGCCAGGTTCTTGGCTTATGT -3' |
| AP001189 | 5'- GTCCACAGGATCAACCGCT -3' | 5'- AAAGTCCCAGGAAGACTGTGC -3' |
| GAPDH | 5'-AAATCAAGTGGGGCGATGCT-3' | 5'-CAAATGAGCCCCAGCCTTCT-3' |

**Supplementary Table S2**. The expression data of enrolled immune-related lncRNAs in SCLC.

| ID | ENOX1-AS1 | AC005162 | LINC00092 | RPL34-AS1 | AC104135 | AC015971 | AC126544 | AP001189 | 8-IrlncRNA |
| --- | --- | --- | --- | --- | --- | --- | --- | --- | --- |
|  |  |  |  |  |  |  |  |  |  |
| 1 | 1.745298 | 2.060457 | 2.615043 | 1.130646 | 2.685949 | 3.384387 | 3.545507 | 3.509715 | 1.348224 |
| 2 | 1 | 1 | 1 | 1 | 1 | 1 | 1 | 1 | 0.6401 |
| 3 | 0.005555 | 2.171888 | 0.017543 | 0.001037 | 1.75E-10 | 0.276832 | 0.861886 | 0.375551 | 0.398138 |
| 4 | 0.015987 | 2.815705 | 0.329961 | 0 | 8.96E-07 | 0.815844 | 0.883721 | 2.628961 | 0.378814 |
| 5 | 0.024269 | 1.105585 | 0.042713 | 0.001231 | 0.059439 | 0.247528 | 0.740347 | 0.229379 | 0.267033 |
| 6 | 0 | 1.936975 | 0.161136 | 1.13E-08 | 0 | 0.291905 | 0.706148 | 0.938851 | 0.25343 |
| 7 | 0.004051 | 1.78526 | 0.056797 | 0.001494 | 0.150807 | 0.107123 | 0.486573 | 0.350036 | 0.239022 |
| 8 | 0.008997 | 2.019105 | 1.04E-09 | 0.003661 | 0.008576 | 0.056122 | 0.095166 | 0.259941 | 0.233147 |
| 9 | 0.004669 | 1.545475 | 0.083824 | 3.2E-16 | 0.098689 | 0.150227 | 0.909226 | 1.115527 | 0.209777 |
| 10 | 8.94E-05 | 1.477436 | 0.043623 | 5.13E-15 | 0.078215 | 0.154098 | 0.593148 | 0.789675 | 0.208766 |
| 11 | 0.002284 | 0.988157 | 0.017714 | 0.000622 | 0.10457 | 0.099213 | 0.538567 | 0.092461 | 0.19053 |
| 12 | 0.001814 | 1.066594 | 0.005524 | 0.000615 | 0.029478 | 0.055744 | 0.216121 | 0.032624 | 0.154772 |
| 13 | 0.006319 | 0.170647 | 0.069197 | 0.097457 | 0.003291 | 0.081637 | 1.084864 | 0.01592 | 0.152829 |
| 14 | 0.004363 | 0.610306 | 0.030315 | 2.3E-05 | 0.202211 | 0.159657 | 0.300718 | 0.260847 | 0.134617 |
| 15 | 2.85E-08 | 0.991029 | 0.021181 | 4.24E-13 | 0.013649 | 0.057307 | 0.322072 | 0.275192 | 0.133756 |
| 16 | 0.002429 | 0.658056 | 0.004546 | 0.000518 | 0.017866 | 0.051439 | 0.389912 | 0.006 | 0.130465 |
| 17 | 0.006685 | 0.538111 | 0.029929 | 0.000782 | 0.146538 | 0.136664 | 0.293865 | 0.066793 | 0.129653 |
| 18 | 0 | 0.198032 | 1.23E-09 | 0.018935 | 0.018952 | 0.160069 | 0.381462 | 0.070655 | 0.122943 |
| 19 | 0.001437 | 0.398622 | 0.007547 | 0 | 0.016836 | 0.093429 | 0.373337 | 0.019505 | 0.114389 |
| 20 | 0.00899 | 0.488363 | 0.010952 | 1.09E-11 | 0.008709 | 0.095428 | 0.338359 | 0.133018 | 0.114228 |
| 21 | 5.03E-05 | 0.310366 | 0.055033 | 1.63E-09 | 0.049055 | 0.097415 | 0.852602 | 0.356271 | 0.113317 |
| 22 | 0.002955 | 0.462168 | 0.007938 | 0 | 0.033438 | 0.059072 | 0.382704 | 0.013225 | 0.110304 |
| 23 | 0.068347 | 0.28242 | 0.085838 | 0.053924 | 0.952148 | 0.110031 | 0.12903 | 0.017653 | 0.110216 |
| 24 | 0.03301 | 0.466513 | 0.028208 | 0 | 0.03293 | 0.13452 | 0.619221 | 0.890677 | 0.107922 |
| 25 | 0.015232 | 0.127564 | 0.193855 | 0.000311 | 0.008288 | 0.275379 | 0.950643 | 0.39165 | 0.107721 |
| 26 | 0.000396 | 0.433547 | 3.92E-10 | 6.85E-10 | 0.044673 | 0.069932 | 0.308322 | 0.016714 | 0.106464 |
| 27 | 0.043069 | 0.273144 | 0.01271 | 0.071032 | 0.1311 | 0.121726 | 0.038401 | 0.025748 | 0.105724 |
| 28 | 0.014677 | 0.321528 | 0.0492 | 0 | 0.123468 | 0.251211 | 0.420997 | 0.937364 | 0.099046 |
| 29 | 0.014985 | 0.234846 | 0.087687 | 0.008937 | 0.023484 | 0.306571 | 0.514575 | 0.994915 | 0.097307 |
| 30 | 9.25E-11 | 0.547026 | 7.41E-05 | 3.62E-14 | 0.017303 | 0.041216 | 0.21955 | 0.081107 | 0.092612 |
| 31 | 3.84E-15 | 0.531331 | 4.17E-13 | 5.95E-11 | 0.027571 | 0.06502 | 0.184453 | 0.151745 | 0.091976 |
| 32 | 0.002225 | 0.5997 | 0.002568 | 0.000468 | 0.033489 | 0.040122 | 0.104642 | 0.013978 | 0.090143 |
| 33 | 0.000543 | 0.479891 | 0.004661 | 0.000406 | 0.043794 | 0.05365 | 0.164101 | 0.01897 | 0.087365 |
| 34 | 0.000387 | 0.338113 | 0.002082 | 0.00024 | 0.060561 | 0.04831 | 0.286979 | 0.004156 | 0.086148 |
| 35 | 0.00485 | 0.382848 | 0.071918 | 0 | 0.091961 | 0.10693 | 0.455311 | 0.222858 | 0.085898 |
| 36 | 0.000486 | 0.37632 | 0.017746 | 0.000701 | 0.099578 | 0.054945 | 0.286446 | 0.052866 | 0.083321 |
| 37 | 0.013011 | 0.161317 | 0.008642 | 0.002232 | 0.019628 | 0.085693 | 0.372351 | 0.140856 | 0.082264 |
| 38 | 0.003966 | 0.397229 | 5.07E-07 | 3.57E-10 | 0.032901 | 0.073605 | 0.444163 | 0.671968 | 0.075944 |
| 39 | 0.001807 | 0.307738 | 0.030897 | 0.001461 | 0.076798 | 0.076987 | 0.339021 | 0.271894 | 0.069115 |
| 40 | 2.4E-09 | 0.274675 | 1.49E-07 | 2.26E-11 | 0.036372 | 0.056206 | 0.228254 | 0.1114 | 0.069017 |
| 41 | 0.000611 | 0.35512 | 0.070128 | 0.074457 | 0.022053 | 0.033045 | 0.35694 | 0.020121 | 0.068973 |
| 42 | 0.025873 | 0.013937 | 0.001021 | 0 | 0.034137 | 0.246786 | 0.507915 | 1.405747 | 0.067209 |
| 43 | 0.006188 | 0.003141 | 0.132357 | 0.006353 | 1.38E-07 | 0.290502 | 0.811623 | 1.112986 | 0.064607 |
| 44 | 0.006945 | 0.223491 | 0.026601 | 0.065753 | 0.135006 | 0.079634 | 0.012893 | 0.016291 | 0.061452 |
| 45 | 0.000385 | 0.065242 | 0.001368 | 0 | 0.031126 | 0.018509 | 0.36289 | 0.010989 | 0.052772 |
| 46 | 2.18E-14 | 0.133732 | 0.123489 | 0 | 0.022867 | 0.205465 | 0.547371 | 0.626825 | 0.051366 |
| 47 | 0 | 0.259948 | 0 | 0.045503 | 0 | 0.037951 | 0.008565 | 0.008923 | 0.051284 |
| 48 | 0 | 0.050781 | 0.042322 | 2.88E-15 | 0.018294 | 0.074156 | 0.341954 | 0.038871 | 0.048186 |
| 49 | 0.000139 | 0.191354 | 0.007027 | 0 | 0.06955 | 0.031557 | 0.162518 | 0.028958 | 0.046902 |
| 50 | 0.001458 | 0.334331 | 0.022644 | 0.000409 | 0.056519 | 0.022074 | 0.162941 | 0.095246 | 0.046702 |
| 51 | 0.000553 | 0.077205 | 0 | 0.04891 | 0.001544 | 0.036627 | 0.132483 | 0.018709 | 0.044939 |
| 52 | 0.00152 | 0.317191 | 0.009542 | 0.035854 | 0.02118 | 0.019096 | 0.010208 | 0.013341 | 0.04481 |
| 53 | 0.001633 | 0.171362 | 0.00678 | 0.054425 | 0.000615 | 0.03838 | 0.023798 | 0.001389 | 0.043256 |
| 54 | 0.008079 | 0.228052 | 0.072524 | 1.36E-09 | 0.05085 | 0.071672 | 0.205763 | 0.007987 | 0.042965 |
| 55 | 0.00473 | 0.157126 | 0.011985 | 0.05108 | 0.027611 | 0.040628 | 0.037163 | 0.006836 | 0.042544 |
| 56 | 0.000124 | 0.059147 | 0.005743 | 0.000139 | 0.022688 | 0.030024 | 0.244421 | 0.004888 | 0.041769 |
| 57 | 0.00368 | 0.253929 | 0.056834 | 0.000272 | 0.023494 | 0.036586 | 0.376894 | 0.247277 | 0.039833 |
| 58 | 0.000104 | 0.015351 | 0.018239 | 0.000435 | 0.081168 | 0.037998 | 0.295946 | 0.034906 | 0.03967 |
| 59 | 0.000323 | 0.141925 | 0.002518 | 8.21E-05 | 0.026745 | 0.018772 | 0.133777 | 0.001561 | 0.036333 |
| 60 | 0.003128 | 0.184816 | 0.088327 | 0.091358 | 0.218263 | 0.08076 | 0.028299 | 0.012796 | 0.03625 |
| 61 | 0.001328 | 0.212403 | 0.283096 | 0.139295 | 0.002595 | 0.119084 | 0.729199 | 0.028707 | 0.03585 |
| 62 | 0.000896 | 0.202166 | 0.028264 | 0.061889 | 0.002877 | 0.020695 | 0.073788 | 0.012055 | 0.035316 |
| 63 | 0.003917 | 0.2409 | 0.068443 | 0 | 0.033065 | 0.037364 | 0.282259 | 0.075625 | 0.034759 |
| 64 | 0.000131 | 0.071246 | 0.017367 | 0.041634 | 0.000319 | 0.030236 | 0.154413 | 0.012556 | 0.034659 |
| 65 | 0.000322 | 0.13134 | 0.00389 | 0.000267 | 0.070649 | 0.01951 | 0.1132 | 0.004668 | 0.033895 |
| 66 | 0.004086 | 0.038376 | 0.00508 | 0 | 0.028524 | 0.025557 | 0.270338 | 0.144815 | 0.033259 |
| 67 | 0.000111 | 0.051631 | 0.00363 | 0.000294 | 0.065228 | 0.019551 | 0.184698 | 0.003676 | 0.033065 |
| 68 | 0.000394 | 0.094601 | 0.010551 | 0.000125 | 0.018164 | 0.033032 | 0.220077 | 0.148264 | 0.032171 |
| 69 | 0.000554 | 0.201019 | 0.013414 | 0.043484 | 0.002172 | 0.01966 | 0.02208 | 0.026269 | 0.031739 |
| 70 | 0.001818 | 0.035429 | 0.007965 | 0.000111 | 0.014729 | 0.027052 | 0.200845 | 0.033511 | 0.030809 |
| 71 | 0.000282 | 0.126215 | 0.029476 | 0.000199 | 0.029448 | 0.033499 | 0.157977 | 0.001067 | 0.030061 |
| 72 | 0.001113 | 0.055998 | 0.057882 | 0.092639 | 0.034314 | 0.068146 | 0.075704 | 0.030268 | 0.029781 |
| 73 | 0.002662 | 0.076328 | 0.018861 | 0.036955 | 0.008229 | 0.045003 | 0.053611 | 0.011854 | 0.029638 |
| 74 | 0.002226 | 0.053342 | 1.09E-05 | 0 | 0.007584 | 0.030645 | 0.086135 | 0.004942 | 0.027389 |
| 75 | 0.008827 | 0.144749 | 0.089423 | 0.08327 | 0.078134 | 0.077617 | 0.043284 | 0.021277 | 0.027211 |
| 76 | 0.000718 | 0.21969 | 0.04697 | 0.058856 | 0.003374 | 0.031984 | 0.026691 | 0.013238 | 0.026727 |
| 77 | 0.00455 | 0.021561 | 0.064144 | 0.058333 | 0.006401 | 0.068008 | 0.176722 | 0.043903 | 0.026696 |
| 78 | 0.000453 | 0.048165 | 0.002044 | 0 | 0.026953 | 0.026406 | 0.112952 | 0.021384 | 0.026013 |
| 79 | 0.001014 | 0.154872 | 0.024562 | 0.040567 | 0.047197 | 0.025598 | 0.01872 | 0.010261 | 0.025535 |
| 80 | 0.004828 | 0.105194 | 0.034665 | 0.050386 | 0.002877 | 0.045083 | 0.012338 | 0.006921 | 0.024234 |
| 81 | 0.000729 | 0.021966 | 3.98E-08 | 0.009374 | 0.045978 | 0.04665 | 0.001038 | 0.001074 | 0.023846 |
| 82 | 0.001285 | 0.074987 | 0.001146 | 0.015667 | 0.010037 | 0.025374 | 0.02914 | 0.003824 | 0.023843 |
| 83 | 0.001136 | 0.116552 | 0.026948 | 0.024009 | 0.01657 | 0.032064 | 0.057134 | 0.002605 | 0.023263 |
| 84 | 0.012904 | 0.314042 | 0.057003 | 0.000891 | 0.043625 | 0.128561 | 0.275494 | 1.026919 | 0.022799 |
| 85 | 0.007104 | 0.045291 | 0.058683 | 0.096801 | 0.102665 | 0.052919 | 0.018189 | 0.013402 | 0.022606 |
| 86 | 0.007878 | 0.147646 | 0.032155 | 0.060283 | 0.004895 | 0.018856 | 0.009018 | 0.008441 | 0.022534 |
| 87 | 0.002086 | 0.108501 | 0.01762 | 0.027855 | 0.027901 | 0.03137 | 0.004281 | 0.005692 | 0.022214 |
| 88 | 0.002019 | 0.046253 | 0.01357 | 0 | 0.045151 | 0.023681 | 0.117922 | 0.010228 | 0.021663 |
| 89 | 0.001367 | 0.083591 | 0.010123 | 0.041187 | 0.033399 | 0.020721 | 0.0071 | 0.009866 | 0.02162 |
| 90 | 0.002896 | 0.12585 | 0.016464 | 1.36E-11 | 0.040052 | 0.029927 | 0.086698 | 0.107844 | 0.021424 |
| 91 | 0.00183 | 0.035283 | 0.041287 | 0.043637 | 0.034284 | 0.044247 | 0.104556 | 0.022852 | 0.02078 |
| 92 | 0.001744 | 0.105462 | 0.016372 | 0.027066 | 0.032489 | 0.016787 | 0.036187 | 0.005445 | 0.02022 |
| 93 | 0.000144 | 0.058894 | 0.004883 | 0.000112 | 0.044064 | 0.015548 | 0.072802 | 0.002202 | 0.01906 |
| 94 | 0.000453 | 0.014029 | 0.022884 | 0.028893 | 0.001263 | 0.049289 | 0.033818 | 0.002608 | 0.018622 |
| 95 | 0.000567 | 0.071589 | 0.00338 | 0.019013 | 0.036794 | 0.01264 | 0.023725 | 0.002852 | 0.018276 |
| 96 | 0.002031 | 0.033393 | 0.040266 | 0.026279 | 0.020382 | 0.064356 | 0.030506 | 0.013718 | 0.01769 |
| 97 | 0.000366 | 0.073161 | 0.003933 | 0.027466 | 0.013168 | 0.011376 | 0.003938 | 0.00106 | 0.016471 |
| 98 | 0.001018 | 0.129656 | 0.030479 | 0.048917 | 0.005372 | 0.016641 | 0.01319 | 0.007235 | 0.016424 |
| 99 | 0.000161 | 0.026779 | 0.002074 | 0.00011 | 0.020333 | 0.006407 | 0.10607 | 0.001248 | 0.016359 |
| 100 | 1.8E-09 | 4.78E-06 | 0.042772 | 0.044747 | 0.022323 | 0.057152 | 0.051217 | 0.011365 | 0.015454 |
| 101 | 0.000458 | 0.026646 | 0.01433 | 3.86E-07 | 0.012662 | 0.039622 | 0.038876 | 0.007929 | 0.01535 |
| 102 | 0.000757 | 0.130346 | 0.038631 | 0 | 0.04884 | 0.042103 | 0.10065 | 0.157003 | 0.013636 |
| 103 | 6.94E-11 | 0.00976 | 0.008995 | 9.81E-13 | 0.021244 | 0.013916 | 0.104466 | 0.00525 | 0.01361 |
| 104 | 0.002021 | 0.138695 | 0.034877 | 0.02908 | 2.73E-05 | 0.022399 | 0.007905 | 0.010283 | 0.013055 |
| 105 | 0.000364 | 0.006293 | 0.017213 | 0 | 0.008905 | 0.037401 | 0.065415 | 0.017148 | 0.013029 |
| 106 | 0.004316 | 0.155853 | 0.058738 | 0.020357 | 0.123242 | 0.030746 | 0.039201 | 0.016694 | 0.012728 |
| 107 | 0.000873 | 0.032313 | 0 | 0 | 0.010194 | 0.016408 | 0.02559 | 0.013486 | 0.012226 |
| 108 | 0.001239 | 0.023437 | 0.011007 | 0 | 0.013788 | 0.024494 | 0.127511 | 0.142966 | 0.011672 |
| 109 | 0.001825 | 0.084279 | 0.036707 | 0.027715 | 0.035321 | 0.026347 | 0.030676 | 0.00595 | 0.011468 |
| 110 | 0.007101 | 0.091783 | 0.039411 | 0.010605 | 0.039224 | 0.028364 | 0.039155 | 0.008035 | 0.01125 |
| 111 | 0 | 0.026026 | 2.16E-07 | 7.53E-14 | 0.022197 | 0.014665 | 0.024834 | 0.015356 | 0.010761 |
| 112 | 0.000569 | 0.092966 | 0.041708 | 0.031582 | 0.061996 | 0.024084 | 0.023031 | 0.000531 | 0.009823 |
| 113 | 0.002525 | 0.051949 | 0.061693 | 0.064558 | 0.085993 | 0.043044 | 0.017017 | 0.009764 | 0.009737 |
| 114 | 0.000922 | 0.004301 | 0.013603 | 0.018322 | 0.016061 | 0.023796 | 0.015537 | 0.006781 | 0.008657 |
| 115 | 0.002034 | 0.069721 | 0.039688 | 0.032553 | 0.030417 | 0.025916 | 0.027816 | 0.0133 | 0.008377 |
| 116 | 0.0005 | 0.043167 | 0.004538 | 0.013496 | 0.001507 | 0.007094 | 0.004063 | 0.002156 | 0.008255 |
| 117 | 0.004913 | 0.097949 | 0.057682 | 0.031724 | 0.076157 | 0.037272 | 0.015255 | 0.013727 | 0.008082 |
| 118 | 0.000463 | 0.017301 | 0.001588 | 5.12E-07 | 0.012 | 0.00827 | 0.033917 | 0.016503 | 0.007352 |
| 119 | 0.001002 | 0.049894 | 0.02784 | 0.026006 | 0 | 0.021094 | 0.013685 | 0.002231 | 0.006701 |
| 120 | 0.000729 | 0.095388 | 0.052111 | 0.018913 | 0.016694 | 0.027931 | 0.063776 | 0.008627 | 0.006501 |
| 121 | 0.00103 | 0.029961 | 0.019813 | 0 | 0.017786 | 0.019667 | 0.044008 | 0.011522 | 0.006103 |
| 122 | 0.000364 | 0.008276 | 0 | 0 | 0.012993 | 0.007338 | 0.024265 | 0.020911 | 0.005467 |
| 123 | 0.000249 | 0.023088 | 0.004403 | 0 | 0.01355 | 0.004915 | 0.01615 | 0.014553 | 0.003519 |
| 124 | 0.006033 | 0.073007 | 0.039894 | 0.049223 | 0.043479 | 0.001896 | 0.0031 | 0.003617 | 0.002523 |
| 125 | 0.00044 | 0.052259 | 0.028849 | 0.029438 | 0.007045 | 0.008205 | 0.011735 | 0.002964 | 0.001959 |
| 126 | 0.015535 | 0.058794 | 0.066326 | 0.032644 | 0.047004 | 0.034348 | 0.009041 | 0.006494 | 0.001548 |
| 127 | 0.00248 | 0.201775 | 0.083776 | 0.056615 | 0.003817 | 0.016476 | 0.01993 | 0.000572 | 0.001501 |
| 128 | 0.002887 | 0.100829 | 0.065088 | 0.000288 | 0.009185 | 0.03497 | 0.06341 | 0.00541 | 0.00064 |
| 129 | 0 | 1.24E-07 | 3.6E-11 | 5.45E-15 | 0.018765 | 0.002707 | 0.005258 | 0.031815 | 0.000104 |
| 130 | 0.001733 | 0.030689 | 0.084811 | 0.018604 | 0.091904 | 0.047809 | 0.110261 | 0.008302 | -0.0007 |
| 131 | 0.00056 | 0.013115 | 0.040899 | 0.039699 | 0.000219 | 0.012935 | 0.030989 | 0.000354 | -0.00214 |
| 132 | 0.000156 | 0.236045 | 0.098793 | 0.03555 | 0.001475 | 0.027712 | 0.02767 | 0.008084 | -0.00245 |
| 133 | 0.000542 | 0.015076 | 0.053308 | 0.03654 | 0.01301 | 0.029824 | 0.024639 | 0.007505 | -0.00279 |
| 134 | 7.42E-05 | 0.055997 | 0.056158 | 0.022858 | 0.000941 | 0.020791 | 0.055564 | 0.006506 | -0.0031 |
| 135 | 0.000291 | 0.105664 | 0.084436 | 0.055843 | 2.29E-10 | 0.027624 | 0.042344 | 0.010869 | -0.0041 |
| 136 | 0.000195 | 0.042703 | 0.05799 | 0.026254 | 0.006914 | 0.020471 | 0.029989 | 0.006878 | -0.00739 |
| 137 | 0.00038 | 0.043692 | 0.066138 | 0.030335 | 0.019723 | 0.018598 | 0.03473 | 0.01158 | -0.01045 |
| 138 | 0.001948 | 0.016472 | 0.050173 | 0 | 0.009994 | 0.003164 | 0.012943 | 0.009821 | -0.01941 |
| 139 | 0.003853 | 0.400107 | 0.223121 | 0 | 0.009709 | 0.055321 | 0.307212 | 0.260507 | -0.02632 |
| 140 | 0.002148 | 0.068848 | 0.125036 | 0.022799 | 0.094224 | 0.033758 | 0.047566 | 0.010776 | -0.02725 |
| 141 | 0.004774 | 0.178485 | 0.155195 | 0 | 0.010929 | 0.03058 | 0.127122 | 0.006293 | -0.0287 |
| 142 | 0.004488 | 0.065319 | 0.133738 | 0 | 0.011157 | 0.044221 | 0.166473 | 0.173107 | -0.03212 |
| 143 | 0.000405 | 0.049849 | 0.125026 | 0.032251 | 0.00133 | 0.024667 | 0.026071 | 0.008997 | -0.03681 |
| 144 | 0.001451 | 0.051899 | 0.13545 | 0.021895 | 0.002523 | 0.030415 | 0.032961 | 0.006326 | -0.04007 |
| 145 | 0.000537 | 0.027653 | 0.119157 | 0.017439 | 0.007094 | 0.024255 | 0.007159 | 0.008384 | -0.04111 |
| 146 | 0.003873 | 0.126972 | 0.17693 | 0.034241 | 0.004682 | 0.029888 | 0.021609 | 0.005242 | -0.05007 |
| 147 | 0.000162 | 0.009594 | 0.16164 | 0.038698 | 0.001918 | 0.033055 | 0.005847 | 0.005419 | -0.05625 |
| 148 | 0.001175 | 0.203591 | 0.230683 | 0.02988 | 0.007499 | 0.042889 | 0.024206 | 0.011678 | -0.06471 |
